# Editorial: Current challenges in inflammation and pain biology: The role of natural and synthetic compounds

**DOI:** 10.3389/fphys.2022.1008538

**Published:** 2022-09-07

**Authors:** Elizabeth Soares Fernandes, Emer Suavinho Ferro, Gisele Simão, Guilherme Alves de Góis, Jack Arbiser, Soraia Kátia Pereira Costa

**Affiliations:** ^1^ Programa de Pós-graduação em Biotecnologia Aplicada à Saúde da Criança e do Adolescente, Faculdades Pequeno Príncipe, Curitiba, Brazil; ^2^ Instituto de Pesquisa Pelé Pequeno Príncipe, Curitiba, Brazil; ^3^ Departamento de Farmacologia, Universidade de São Paulo, São Paulo, Brazil; ^4^ Veterans Administration Medical Center, School of Medicine, Emory University, Atlanta, NY, United States

**Keywords:** pain, inflammation, mechanisms of disease, natural compounds, synthetic therapies

Inflammation and pain are complex physiological responses which can evolve to chronic disorders. They involve an intricate network of molecules and receptors (Chen et al., 2018; Yam et al., 2018), often making their management, a clinical challenge. The discovery and development of natural and synthetic compounds with analgesic and/or anti-inflammatory activities have highly contributed to halt or attenuate different types of painful and inflammatory diseases (Atanasov et al., 2021; Beck et al., 2022). Their side effects, of special concern in chronic pathologies, prompted an increasing number of non-clinical and clinical studies aiming at evaluating the effectiveness and safety of novel natural and synthetic products for treating inflammation and pain. However, the costs involved in developing and implementing new therapies and the several challenges encountered including low selectivity, high incidence of adverse effects and/or unfavourable pharmacokinetics properties of drug candidates have been major setbacks in the field (Tautermann, 2020). These, highlight the need for novel studies focused on the discovery and development of analgesic and anti-inflammatory drugs. This Research Topic brings together senior researchers, clinicians, and researchers, to share science and hopes to treat inflammatory and painful disorders.

Eight articles were published in the Current Challenges in Inflammation and Pain Biology: The Role of Natural and Synthetic Compounds Research Topic; these have been viewed more than 12,000 times. The articles published in this collection provided new information on diverse pathologies, including nephropathy, dermatitis, rheumatoid arthritis (RA), cerebral ischemia, diabetes, osteogenesis, and neuropathic and abdominal pain. They also explored novel therapeutic strategies for these diseases such as synthetic and natural compounds, as well as the effects of laser photobiomodulation.

Two of the articles were reviews, and one, a mini-review. The review by Campos et al. discussed the importance of the endocannabinoid system in chronic neuropathic pain and the evidence for the use of cannabinoids to treat this disorder. Shang et al. revised the different inflammatory pathways involved in cerebral ischemia and highlighted the potential of major classes of natural compounds in containing damage caused by inflammation in this pathology. The mini-review by Decraecker et al. focused on the physiological roles of proteases and on the emerging use of protease inhibitors as therapies for visceral hypersensitivity and inflammatory bowel disease.

Five original articles were also published in this Research Topic. By using *in vivo* and *in vitro* models, Liu et al. demonstrated that inhibition of NLRP3 inflammasome activation by the natural compound methyl gallate ameliorates hyperuricemia nephropathy. Methyl gallate diminished uric acid production, promoted uric acid excretion, and reduced mouse renal injury. These effects were associated with the ability of the compound to inhibit NLRP3 oligomerization and assembly by impairing reactive oxygen species production by mouse bone marrow-derived macrophages and peripheral blood mononuclear cells (PBMCs) from healthy subjects and patients with gout.

In another very interesting study, Ramirez-Perez et al. demonstrated the effects of ST2825, a synthetic inhibitor of MyD88 dimerization, on PBMCs from RA patients who were naive for therapy with disease-modifying anti-rheumatic drugs. Analysis of RNA-sequencing data indicated that the *in vitro* incubation of ST2825 down-regulates different genes such as those involved in the complement and matrix metalloproteinases pathways, and chemokine signalling in RA PBMCs. They also suggested ST2825 can potentially modulate these genes in synovial cells of arthritic patients, and highlighted the compound as an emerging therapeutic strategy for RA.

In a report by Li et al., the role of CB2R was investigated in an *in vivo* model of psoriatic dermatitis induced by imiquimod. The authors found increased CB2R protein expression in the epidermis of human psoriatic skin lesions. Then, they used the synthetic CB2R agonist JWH-133 and CB2R knockout and wild type mice to demonstrate that CB2R is an important target for the management of psoriasis-associated itch and inflammation. Proliferation and prolongation of nerve fibres paralleled to high expression of nerve growth factor were observed in psoriatic skin lesions. CB2R deficiency resulted in exacerbated pruritus due to increased expression of pro-inflammatory cytokines and accumulation of CD4^+^ T cells in the inflamed skin. Also, a higher proportion of splenic Th17/Treg cells were noted in mice lacking of CB2R. In contrast, treatment with JWH-133 protected against the disorder, an effect largely prevented by the CBR2 antagonist AM-630.

Ding et al. assessed the anti-diabetic actions of astilbin, a natural occurring flavonoid. It was found that this compound ameliorates type-1 diabetes in NOD mice, reducing hyperglycemia and preventing weight loss. This effect was associated with less infiltration of CD4^+^ T cells in the pancreas. TNFα and IFNγ release by CD4^+^ T cells was markedly impaired by astilbin. At molecular level, the effects of this flavonoid on CD4^+^ T cells were due to PPARγ activation and downstream down-regulation of STAT3, NF-κB, Akt, p38 MAPK, and mTOR, in addition to increased expression of SOCS3 and PTEN.

Finally, *in vitro* irradiation of laser photobiomodulation at 808 nm was found to affect the proliferation, growth and differentiation factors, mineralization, and extracellular matrix remodeling genes in human dental pulp stem cells stimulated with lipopolysaccharide (da Rocha et al.). PAR2-PAR4 genes were up-regulated whilst PAR-1 was down-regulated by the laser therapy. The laser irradiation also markedly attenuated the gene expression of the matrix metalloproteinases 8 and 9, in addition to those of TNFα, bone morphogenetic proteins 1, 4 and 7, and growth factors including FGF1, GDF10, IGF2, TGFβ1 and VEGFB. Furthermore, genes for receptors (vitamin D receptor, FGF receptor) and proteins such as those from the Smad family (Smad 1 and 4) were down-regulated by the therapy. These findings indicate the potential of the laser therapy at infrared wavelength to prevent bone resorption and inflammation, and therefore, protect dental structures.

Despite the need for continuing searching for new therapeutic options, the studies published in this Research Topic present novel therapeutic approaches which might represent future forms of control/cure of inflammation and pain. Therefore, we would like to thank all the authors for their efforts to elucidate mechanisms of action of such therapies, as well as the reviewers who provided important contributions to the published manuscripts.

## References

[B1] Atanasov A. G. Zotchev S. B. Dirsch V. M. Supuran C. T. International Natural Product Sciences Taskforce (2021). Natural products in drug discovery: Advances and opportunities. Nat. Rev. Drug. Discov. 20 (3), 200–216. 10.1038/s41573-020-00114-z PubMed Abstract | 10.1038/s41573-020-00114-z | Google Scholar 33510482 PMC7841765

[B2] Beck H. Härter M. Haß B. Schmeck C. Baerfacker L. (2022). Small molecules and their impact in drug discovery: A perspective on the occasion of the 125th anniversary of the bayer chemical research laboratory. Drug Discov. Today 27 (6), 1560–1574. 10.1016/j.drudis.2022.02.015 PubMed Abstract | 10.1016/j.drudis.2022.02.015 | Google Scholar 35202802

[B3] Chen L. Deng H. Cui H. Fang J. Zuo Z. Deng J. (2018). Inflammatory responses and inflammation-associated diseases in organs. Oncotarget 9 (6), 7204–7218. 10.18632/oncotarget.23208 PubMed Abstract | 10.18632/oncotarget.23208 | Google Scholar 29467962 PMC5805548

[B4] Tautermann C. S. (2020). “Current and future challenges in modern drug discovery,” in Quantum mechanics in drug discovery. Editor Heifetz A. (New York, NY: Humana), 2114. Methods in Molecular Biology. 10.1007/978-1-0716-0282-9_1 10.1007/978-1-0716-0282-9_1 | Google Scholar 32016883

[B5] Yam M. F. Loh Y. C. Tan C. S. Khadijah Adam S. Abdul Manan N. Basir R. (2018). General pathways of pain sensation and the major neurotransmitters involved in pain regulation. Int. J. Mol. Sci. 19, 2164. 10.3390/ijms19082164 10.3390/ijms19082164 | Google Scholar PMC6121522 30042373

